# Pointing Instruments

**Published:** 1898-11

**Authors:** W. H. Steele


ARTICLE VI.

### POINTING INSTRUMENTS.

W. H. STEELE.

Read before the Northern Iowa Dental Society.

Great care should be taken not to overheat steel, as it destroys the grain and makes it brittle. Small instruments should never be heated to a degree that will raise scales while being worked. In drawing a point, all sides must be hammered alike, otherwise the point is liable to spring in tempering, as the side hammered most will have the finest grain.

When a point has once been forged flat, do not attempt to hammer it back to a round or square, as such manipulation will spoil the instrument. Steel should not be hammered after the color fades, until in the final heating, when it should be hammered to a dead black, and the last strokes should be on the cutting edge.

After the instrument is forged to shape, as well as can be with the hammer, it should be dressed up with suitable files, then tempered. For fine tempering, I prefer night or a dark corner, in order to better watch the color.

For drills heat the point to a cherry-red, and dip in mercury. For excavators and small chisels, make a shallow tray one-eighth inch deep, and fill with the following : One ounce of wax, one-half ounce of resin. Heat the point to a deep red and plunge into the composition. This method is preferable to tempering and drawing for small instruments, as it is more certain and requires less skill to produce uniform results.

For larger instruments, I prefer to temper and draw, using a bath made of sperm oil and tallow. Heat to a deep cherry-red, dip the same as in a water bath, and draw to a pale straw color. If a chisel is desired very hard on the cutting edge, place that part of the instrument on a piece of cold steel, hold the shank in the flame, and draw to a straw color, allowing the color to run no farther than to the portion resting on the steel.

After the instruments are tempered they can be finished on the lathe with an Arkansas stone and polished with crocus, using three grades—coarse, medium and fine. Use the first two grades on cotton buff wheels, and the last one on a chamois buff.

Broaches, explorers and other small instruments can be annealed very soft and even by placing them in a piece of iron tubing; fill around with iron or brass filings, plug the ends of the tube, heat it gradually to a red heat, cover over with hot sand, and allow to cool over night.

To temper small springs, heat to a cherry-red and dip into water, then place in a dish and cover with oil; hold the dish over the flame until the oil catches fire, then set away and allow them to cool in the oil.

In tempering in oil or water, always dip the instrument perpendicularly. If dipped on a slant, it is liable to cause the instrument to spring, especially if the bath is very cold. When tempering light work, the instrument must be handled quickly, for the edge where the best temper is required is the smallest part and cools first, often rendering the temper defective just where it should be most perfect.—*Indiana Dental Journal.*

